# Effects and biological limitations of +Gz acceleration on the autonomic functions-related circulation in rats

**DOI:** 10.1007/s12576-016-0461-4

**Published:** 2016-06-04

**Authors:** Yasuhiro Nishida, Satoshi Maruyama, Ichiro Shouji, Takehito Kemuriyama, Akimasa Tashiro, Hiroyuki Ohta, Kohsue Hagisawa, Megumi Hiruma, Hidetake Yokoe

**Affiliations:** 1 Department of Physiology, National Defense Medical College, 3-2 Namiki, Tokorozawa, Saitama 359-8513 Japan; 2 Pharmacochemical Section, Aeromedical Laboratory, Japan Air Self-Defense Force, 1-2-10 Sakae, Tachikawa, Tokyo 190-8585 Japan; 3 Department of Oral and Maxillofacial Surgery, National Defense Medical College, 3-2 Namiki, Tokorozawa, Saitama 359-8513 Japan

**Keywords:** +Gz stress, Arterial pressure at the brain level, Renal sympathetic nerve activity, Cardiac output, Total peripheral resistance, Myocardial troponin, Brain-tissue oxygen level, Brain-tissue blood flow, Teeth-clenching pressor response, Preconditioning

## Abstract

The effects of gravitational loading (G load) on humans have been studied ever since the early 20th century. After the dangers of G load in the vertical head-to-leg direction (+Gz load) became evident, many animal experiments were performed between 1920 and 1945 in an effort to identify the origins of high G-force-induced loss of consciousness (G-LOC), which led to development of the anti-G suit. The establishment of norms and training for G-LOC prevention resulted in a gradual decline in reports of animal experiments on G load, a decline that steepened with the establishment of anti-G techniques in humans, such as special breathing methods and skeletal muscle contraction, called an anti-G straining maneuver, which are voluntary physiological functions. Because the issue involves humans during flight, the effects on humans themselves are clearly of great importance, but ethical considerations largely preclude any research on the human body that probes to any depth the endogenous physiological states and functions. The decline in reports on animal experiments may therefore signify a general decline in research into the changes seen in the various involuntary, autonomic functions. The declining number of related reports on investigations of physiological autonomic systems other than the circulatory system seems to bear this out. In this review, we therefore describe our findings on the effects of G load on the autonomic nervous system, cardiac function, cerebral blood flow, tissue oxygen level, and other physiological autonomic functions as measured in animal experiments, including denervation or pharmacological blocking, in an effort to present the limits and the mechanisms of G-load response extending physiologically. We demonstrate previously unrecognized risks due to G load, and also describe fundamental research aimed at countering these effects and development of a scientific training measure devised for actively enhancing +Gz tolerance in involuntary, autonomic system functions. The research described here is rough and incomplete, but it is offered as a beginning, in the hope that researchers may find it of reference and carry the effort toward completion. The advances described here include (1) a finding that cerebral arterial perfusion pressure decreases to nearly zero under +5.0 Gz loads, (2) indications that G load may cause myocardial microinjuries, (3) detection of differences between cerebral regions in tissue-oxygen level under +3.0 Gz load, (4) discovery that hypotension is deeper under decreasing +Gz loads than increasing +Gz loads with use of an anti-G system, due in part to suppression of baroreceptor reflex, and (5) revelations and efforts investigating new measures to reduce cerebral hypotension, namely the “teeth-clenching pressor response” and preconditioning with slight but repeated G loads.

## Introduction

In accelerating aircraft, pilots are exposed to the force of the acceleration (a “gravitational load”; G load) [1, 41]. In a fast turn, the G load is high. It is exerted strongly downward, from head to legs, especially during rapid turning, and is therefore called a “+Gz load”. It strongly affects the blood flow of the cephalic and aortal arteries toward the lower body, and thus tends to cause marked cephalic hypotension [5] and cerebral ischemia [51] that may result in severe damage to both the brain and the heart. It typically results in loss of consciousness (G-LOC) [4, 10, 52], and loss of visual function in the form of narrowed or darkened vision (gray-out or black-out) [4, 10, 52]. Pilot training typically includes various types of acceleration-load (G-load training) facilities, to increase their G-load resistance or G-tolerance and to prevent physiological damage [1, 7, 8, 41, 47]. In the USA, early forms of acceleration-load training were initiated around 1917 [7], and the modern version emerged in 1980 and was reportedly established as a training course in 1983 [50]. In Japan, acceleration-load training began around 1975 with the training equipment then available. That equipment was replaced with a modernized version in 1980, and that equipment has since been updated and replaced with modern systems [50]. Little detailed information is available on the systems and trends in countries other than the US and Japan. It is said that acceleration-load training in Japan during the past 37 years or so has been developed according to the experience of flight surgeons and by referencing US models. In any case, ensuring safety is the foremost priority in human training, with the highest possible degree of hazard avoidance and implementation performed only in accordance with clearly established programs.

Various acceleration-load (G-load) experiments have been performed throughout the world using animals, but the volume of scientifically researched and published data is smaller than might be expected. PubMed serves as an example. Only about two dozen reports of G-LOC animal experiments can be found in the literature and only half of those concern G-load animal experiments that included the use of anti-G equipment. No doubt, much related research has remained unpublished because it is military-related, but given the 70- or 80-year history of this field, the quantity of scientific data seems unfortunately small.

For over 10 years we have been performing G-load experiments on rats with and without anti-G equipment [31, 32], measured the general circulatory system indicators, cardiac output, sympathetic nerve activity, brain-tissue oxygen level, and other parameters, and applied them to scientific analysis [33, 34]. Well within the limits prescribed by the applicable code of ethics for animal experiments, we have found it possible to observe the state and condition of the subjects without the need for attached safety gear, observe the effects of high G load on the circulatory system, and perform invasive investigations of endogenous functions such as partial physical destruction of physiological functions for identification of active endogenous reflex functions and elucidation of active mechanisms through pharmacological blocking. These results have led to some surprising insights into states and conditions induced by +Gz load, as described in this review.

The review also describes two modes of our basic research on methods of increasing safety under G load. One is focused on the action of teeth clenching, and the research showed for the first time that the pressor response thus instigated can moderate the level of hypotension induced by +Gz loads. In the other, we used brain-tissue oxygen level as an indicator of the effectiveness of preconditioning by repeated exposure to low G loads prior to regular G-training and -norms.

## Small-animal acceleration system

We designed and constructed a two-armed centrifuge for acceleration load and testing of small animals (Tomy Seiko, Tokyo, Japan; [16, 22, 31, 32]). Each arm is 115 cm in length. The laboratory animal is held fixed on a flat table attached to the end of one arm, to obtain gravitational (+Gz) load in the direction from head to tail. Any loading can be selected up to a maximum load capacity of +9.0 Gz (at 86 rpm). At a given load setting, the G onset or offset rates are not linear, but rather exponential, with 0.03 G/second (s) at 3.0 G, 0.04 G/s at 5.0 G, and 0.05 G/s at 7.0 G [22, 31]. The rotational speed of the flat plate is separately measured with a tachometer and recorded, and then converted to the G-value by calculation.

All instruments for measuring arterial pressure (AP) and other parameters are mounted on the centrifuge, with their signals transmitted from the centrifuge via a slip-ring, converted by an analog-to-digital converter, and then recorded to and saved on a computer [16, 22, 31].

The laboratory animals in these experiments were Sprague–Dawley rats (12–16 weeks old, body weight 360–480 g), all of which were under urethane anesthetization throughout the acceleration experiments. All animal experimental procedures were performed in accordance with the guidelines for proper conduct of animal experiments issued by the Science Council of Japan, following approval by the Committee of the Center for Laboratory Animal Science of the National Defense Medical College.

## Effects of G load without anti-G protection

### Effects on cephalic arterial pressure

**Fig. 1:**
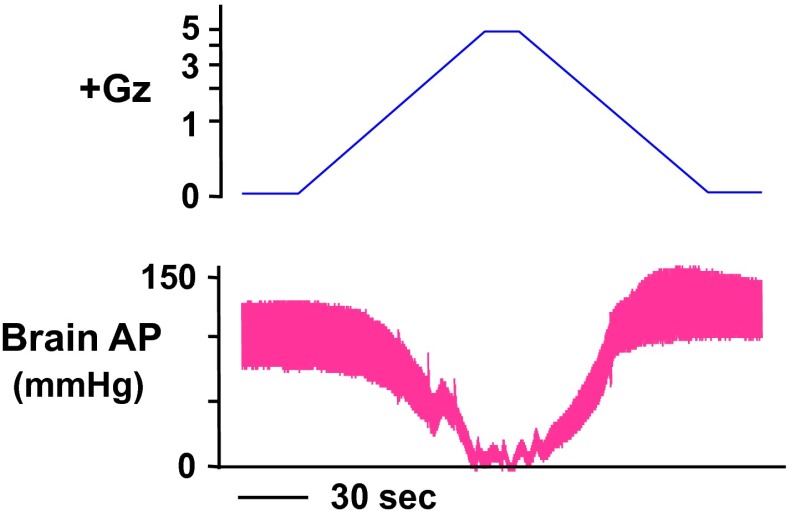
Representative original recordings of arterial pressure at the level of the brain (brain AP) of an intact rat exposed to +5.0 Gz acceleration without anti-G equipment. A Sprague–Dawley rat (350 g body weight) anesthetized with urethane (1.2 g kg^−1^ body weight) was placed in a prone position on the flat table of the centrifuge (115-cm-long arm; see details in text). The front teeth of the rat were fastened to the centrifuge table using a string. An arterial catheter was connected to a pressure transducers (DX-360, Nihon Koden, Tokyo, Japan) placed at the level of the rat brain. A G value was determined using the length of the center-to-head position of the animal and revolution speed at the animal’s head. The ordinate axis of loaded Gz is expressed as a logarithmic scale. When the rat was exposed to +5.0 Gz (head-to-tail longitudinal G) for 15 s at an on-set rate of 0.004 G/s), brain AP was decreased to around a zero level. The mean values of seven rats were shown in the text [22, 25, 31–34]

A marked decline in cephalic AP under +G acceleration occurred in the anesthetized rats without anti-G protection. Under a load of +5.0 Gz, as shown in Fig. 1, cephalic AP was approximately zero, which is a decline of approximately 80 mmHg from before +Gz load to −4 ± 10 mmHg (*n* = 7; SD). This means that at this near-zero cephalic AP, the blood flow to the brain is also near zero. Under a load of +1.5 Gz, the cephalic AP declined by approximately 20 to 60 ± 8 mmHg (*n* = 7; SD), and under a load of +3.0 Gz it declined by approximately 50 to 24 ± 16 mmHg (*n* = 7; SD) [17, 22, 25]. Under a load of +7.0 Gz, the high mortality rate precluded the collection of enough data to allow further statistical analysis.

The cephalic AP generally increased rapidly toward recovery as the Gz load gradually decreased from the 15-s peak +5.0 Gz load. The slope of this increase (change in AP over change in G) was steeper than that of the AP decline during the increase in +Gz load, and thus the AP was approximately 20 mmHg higher than that of the control (sham G load) when the Gz load reached zero. This may be attributable to increased sympathetic nerve activity and secretion of catecholamine and other humoral factors in response to decreasing BP and thus a substantially greater increase in peripheral vascular resistance (VR) under increasing +Gz than decreasing +Gz, but the hormonal analysis has not yet be completed.

It is important to note that under anesthesia, there is little or no motion of the skeletal muscles other than those used for respiration. The experimental results thus suggest that if fighter pilots and anti-G trainees undergo unmitigated G load (sometimes as high as +9 to +11 Gz) without performing the routine techniques of an anti-G straining maneuver [1, 41] then at +5.0 Gz, their cephalic arterial perfusion pressure will decrease to nearly zero, with a significant effect on cerebral blood flow. Even at +3.0 Gz, the cephalic arterial perfusion pressure will decrease to one-third the normal pressure or lower, with a large effect on brain and other cephalic functions. The results also suggest that in the recovery from this deep hypotension, the heart load (oxygen consumption) is stronger during G relaxation than during G onset, in view of the sharp increase in BP during that period as compared with the decrease in BP during the onset.

Peterson et al. [43] have reported that the aortic pressure at the aortic root decreases to 67 ± 13 mmHg in α-chloralose anesthetized dogs when exposed to a +3.0 Gz load and then increases to 168 mmHg following the exposure. Finally, Burns et al. [2] have reported that in anesthetized minipigs the eyeball-level AP decreases to −17 ± 15 mmHg when exposed to +5.0 Gz load. Reports on measurement of AP in the head region of laboratory animals when exposed to +Gz load without the use of anti-G protective equipment are few in number, but none of the prior data conflict with the measured values and changes found in our experiments.

### Effects on sympathetic nerve activity

It is generally well known that when AP is ramped down (gradually decreased) by continuous bleeding, peripheral sympathetic nerve activity is gradually increased by baroreceptor reflex [9, 35]. The nerve activity stops increasing when the mean AP nears 50 mmHg, however, and then begins to decrease with any further decline in AP. The AP then begins a further rapid decline due to the decreased sympathetic nerve activity, accompanied by a roughly simultaneous complete loss of the peripheral sympathetic nerve activity [30]. The exact mechanism is not known in detail, but it is thought to be a “Resignation Response” mediated by endogenous opioids [30]. There have been no previous reports on measurements of sympathetic nerve activity under G load, but we suspected that because of the marked decrease in AP under +Gz load observed, it is plausible that a ‘Resignation Response’ is occurring in the peripheral sympathetic nervous system. We therefore managed to measure the peripheral sympathetic nerve activity under +Gz load by applying a method for recording the renal sympathetic nerve activity (rSNA) [35, 36].

**Fig. 2:**
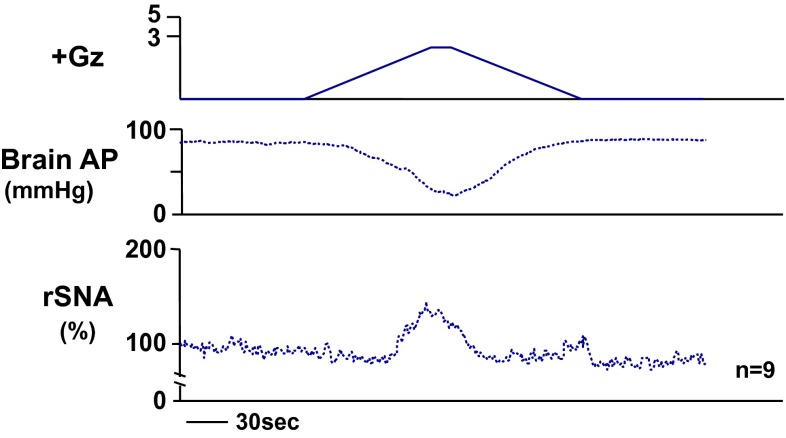
The mean responses of renal sympathetic nerve activity (rSNA) and arterial pressure at the level of the brain (brain AP) to +3.0 Gz acceleration in nine rats without anti-G equipment. rSNA was recorded from the postganglionic renal nerve using stainless-steel electrodes (AS633, Cooner Wire, Chatsworth, CA, USA) fixed by silicone gel (SIL604S-A and -B, Kagawa-Kikai, Japan) in rats anesthetized with urethane as described in Fig. 1. The background noise was determined by enough hypertension to suppress RSNA with phenylephrine. Nerve activity was expressed in %, calculated as [RSNA activity × 100/resting RSNA level observed in the control period]. Refer details on nerve recordings in [22, 35, 36]. The loaded Gz is expressed as a logarithmic scale. Acceleration of +3.0 Gz without anti-G equipment decreased brain AP from 90 ± 3 to 23 ± 11 mmHg, and increased rSNA from 100 to 142 ± 31 %. The peak time of increase in rSNA was somewhat earlier than that of deepest decline in brain AP, indicating that rSNA was beginning to decrease, but did not reach to a zero level, which indicates not a ‘Resignation Response’ phenomenon (see in text) [17, 22, 25, 30]

We found, contrary to our expectation, that under +3.0 Gz load the rSNA increased from approximately 100–142 ± 31 % as cephalic AP decreased from approximately 90–23 ± 11 mmHg (Fig. 2) [17, 22, 25]. Rather than decreasing to zero, the sympathetic nerve activity thus actually kept increasing. We did observe, however, that the sympathetic nerve activity had already passed its peak value and was beginning to decline somewhat at the time of deepest decline in AP (the time of its minimum value). This decline may have been caused by AP descent to a level below the baroreflex activation range for AP (generally about 50–150 mmHg) [9], but this explanation has not been confirmed. It is also possible that the abovementioned ‘Resignation Response’ was just beginning. That said, we have not investigated the effects of opioids or receptor blockers, and the mechanism therefore remains uncertain.

We also measured the rSNA under +5.0 Gz load, but found that the nerve activity initially increased but thereafter oscillated widely in some cases and continued to increase in others, and we were thus unable to obtain a consistent response. These results lead to our speculation that the response, including the brain ischemic response, is highly complex and differs between individuals depending on their natures when the brain-level AP declined to near zero.

In summary, the results showed that peripheral sympathetic nerve activity increases under +Gz load and further that it is suppressed somewhat at strong +Gz loads of +3.0 Gz or more, the ‘Resignation Response’ leading to zero activity does not occur in the range tested. We have not been able to find any previous reports involving measurement of sympathetic nerve activity under +Gz load, and our research in this area is apparently the first that has been made public.

### Effects on the heart

**Fig. 3:**
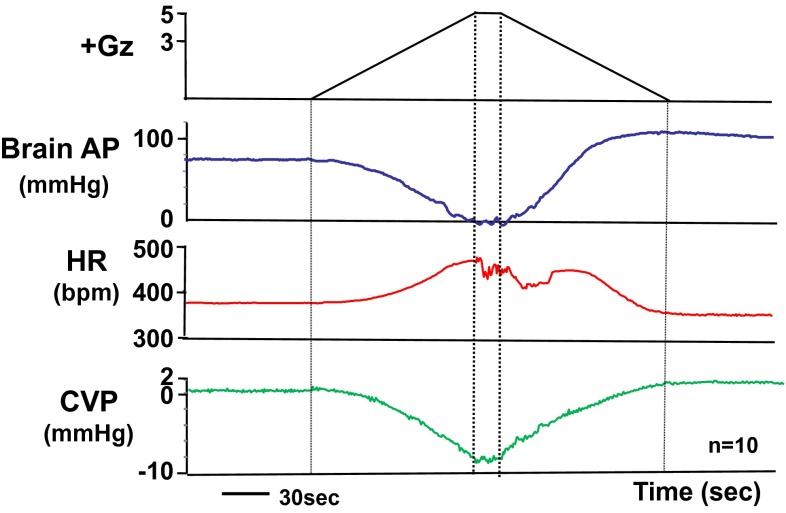
The mean responses of heart rate (HR), central venous pressure (CVP), and arterial pressure at the level of the brain (brain AP) to +5.0 Gz acceleration in ten rats without anti-G equipment. HR was determined using a cardiotachometer coupled triggered by the R wave of the electrocardiogram. A CVP catheter was inserted from the right jugular vein and the catheter tip was put in the superior vena cava. The pressure transducer was fixed at the level of the rat heart. The loaded G is expressed as a logarithmic scale. Acceleration of +5.0 Gz without anti-G equipment decreased CVP from around a zero to −10 ± 1 mmHg, and increased HR from around 380–471 ± 20 bpm. CVP became a marked negative pressure, and arrhythmia occurred during the peak of +5.0 Gz and the G-decreasing phase [15, 16]

Under +5.0 Gz load, the cephalic AP declined to near-zero, central venous pressure (CVP) declined to −10 ± 1 mmHg, and heart rate increased to 471 ± 20 bpm (*n* = 10; SD) (Fig. 3). The rate of change in the magnitude of CVP was about the same during increasing and decreasing +Gz load, and CVP was also about the same before and after the +Gz load (0.7 ± 1.1 and 1.2 ± 1.0, respectively; *n* = 13; SD) [15, 16]. The pressure change was approximately symmetrical between the left and right of a vertical line through the center of the +5.0 Gz load (Fig. 3), which indicates that the rate of venous return varies in a roughly uniform, mechanical correspondence with the amount of +Gz load [15, 16].

The heart rate, in contrast, showed the onset of arrhythmic bradycardia a few seconds after attainment of the +5.0 Gz load and continued for some time thereafter. The bradycardia further intensified when the period (15 s) of +5.0 Gz load ended and the *G* value began to decrease, but the heart rate then gradually became more regular, returning to normal and regaining the same level as the control (sham +Gz load) in correspondence with the continuing decline in +Gz load. The measured CVP values under the +5.0 Gz load indicate that a substantial decrease in cardiac output occurred and furthermore, that the diastolic AP (which drives coronary blood flow) also declined, causing a transient decline in myocardial blood flow. These effects, taken together with the observed increase in AP and consequent increase in myocardial oxygen demand on relaxation of the +Gz load, suggest that myocardial ischemia intensifies while under the +5.0 Gz load and again on its relaxation.

**Fig. 4:**
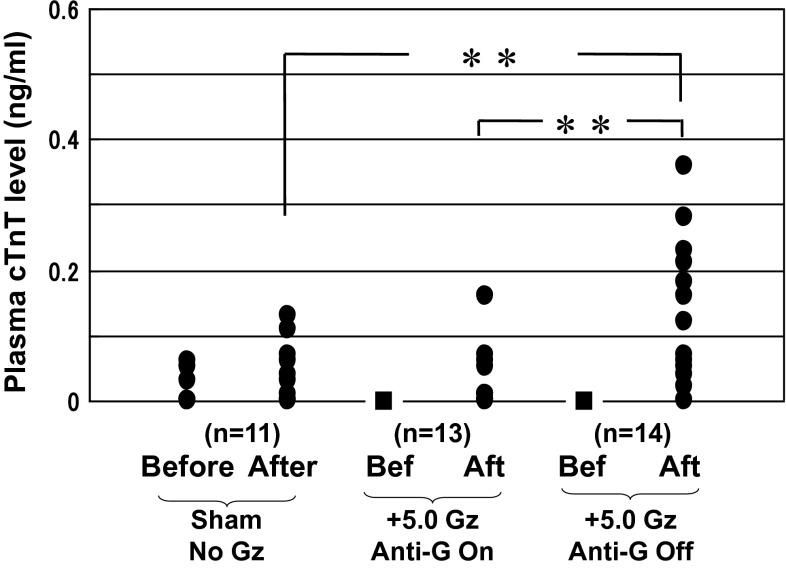
Effects of +5.0 Gz acceleration on the plasma concentration of cardiac troponin T (cTnT) in rats with (*n* = 13) and without (*n* = 14) anti-G equipment (Ant-G). More than a week prior to the experiment, an arterial catheter was implanted aseptically and plugged out with heparinized saline and a metal plug. A blood sample was obtained before and 3 h later after the centrifuge (+5.0 Gz during 15 s) or sham centrifuge. The plasma cTnT level was determined by the electrochemiluminescence immunoassay method. *Filled circle* obtained values, *filled square* not detectable levels, *Before and Bef* before acceleration, *After and Aft* after acceleration. **p < 0.01 by one-factor ANOVA with the Tukey–Kramer test. Significant difference was detected in mean plasma cTnT levels between the ‘After’-of-the-sham-centrifuge group and the ‘After’-of- +5.0 Gz-without Anti-G group (0.04 ± 0.05 vs. 0.14 ± 0.11 ng/ml), and between the ‘After’-of-the- +5.0 Gz-with-Anti-G group and the ‘After’-of- +5.0 Gz-without-Anti-G’ group (0.03 ± 0.05 vs. 0.14 ± 0.11 ng/ml). The data shows that +5.0 Gz acceleration increased plasma cTnT level when without anti-G equipment, but did not when with anti-G equipment, indicating that +5.0 Gz acceleration without anti-G equipment may leave some microinjuries in the myocardium [15, 16]

We investigated the plasma level of cardiac troponin T (cTnT), a specific tissue-indicator of the myocardium, as a marker of myocardial injuries. Figure 4 shows the difference in plasma cTnT level between before and after +5.0 Gz load. Even when compared with when the anti-G system was used, or when loading was not applied, there was a significant increase in plasma cTnT level. The result indicates that the +5.0 Gz load for 15 s without anti-G protection caused myocardial cell injuries. The findings were re-confirmed by optical and electron microscopy examinations [15, 16]. Morphological examinations showed hemorrhage, myofibril rupture, and/or mitochondrial disruption in the myocardium of all of eight rats examined [15, 16]. In summary, the results showed that +5.0 Gz load without an anti-G system has the potential to cause myocardial damage.

Burton et al. [3] applied Gz load to pigs and investigated its morphological effect on cardiac tissue. Following exposure of a +9.0 Gz load for 90 s, they observed gross and microscopic hemorrhaging in the left ventricular myocardium, papillary muscle, and right ventricular myocardium, but found no evidence of hemorrhage after exposure of +9.0 Gz for 45 s with use of a G-suit. The Gz load is substantially larger than the +5.0 Gz applied in our experiments, but the tendencies shown by their experiments with and without use of an anti-G system are in accord with our data.

To investigate the degree of this myocardial damage effect on cardiac function, we measured and compared the cardiac output concurrently with the increase in plasma cTnT level. To measure the cardiac output, we first implanted a blood flow probe on the ascending aorta of the rat under aseptic conditions and performed the measurement approximately 2 weeks later, when no observable surgical effects remained [15, 16]. Comparison of the measurement results before and after +5.0 Gz load showed no effect on cardiac output, either with or without use of an anti-G system. These results indicate that any myocardial damage occurred was insufficient to affect cardiac output, which is an indicator of cardiac function, and therefore slight in degree.

Our reported data were obtained after the second of twice enforcement of +5.0 Gz load for 15 s separated by an interval of 30 min. We have not yet investigated the post-damage course (e.g., recovery, restoration, and cicatrization) following the slight myocardial damage caused by the +5.0 Gz load, myocardial changes under repeated damage, or other effects along a longer-term temporal axis, which therefore remain to be studied. We have investigated +3.0 Gz load for a small number of cases (*n* = 4), however, and found that it had no effect in terms of changes in plasma cTnT level or morphology.

### Effects on the brain

**Fig. 5:**
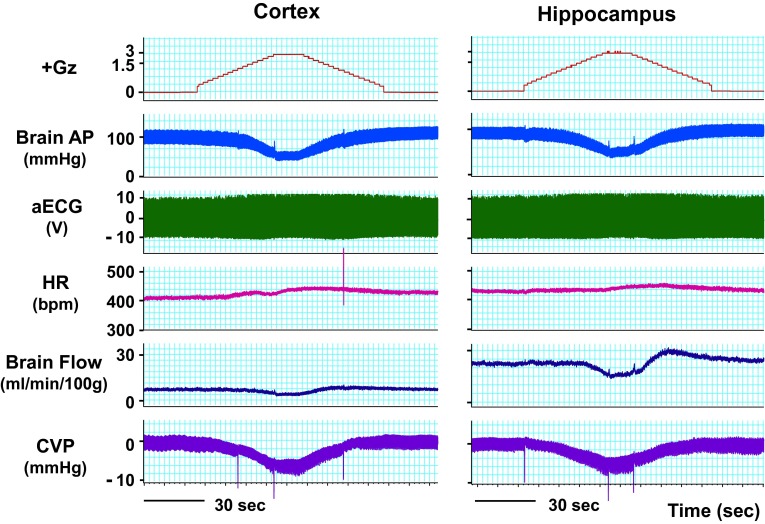
Representative original recordings of brain-tissue blood flow (brain flow) of the cortex or the hippocampus of each rat exposed to +3.0 Gz acceleration for 15 s without anti-G equipment. Brain Flow was measured by the laser flowmeter (One Channel of Omega FLO-C1, Omega-Wave, Tokyo) with a glass-fiber flow probe inserted directly to the brain tissue of the cortex or the hippocampus. The loaded G is expressed as a logarithmic scale. *Brain AP* arterial pressure at a level of the brain, *aECG* amplified electrocardiogram, *HR* heart rate, *CVP* central venous pressure. Acceleration of +3.0 Gz for 15 s without anti-G equipment decreased cortex-tissue blood flow from 5.7 ± 1.9 to 4.3 ± 2.3 ml/min/100 g of tissue, that is 76 ± 9 % (*n* = 6; SE), and hippocampus-tissue blood flow from 19.6 ± 8.1 to 13.5 ± 6.6 ml/min/100 g of tissue, that is 69 ± 6 % (*n* = 7, SE). Although there was no significant difference in %-decrease in blood flow between the two brain tissues, the %-decreases in flow in ml/min/100 g of tissue of the hippocampus (6.1 ± 2.4) was significantly larger than that of the cortex (1.4 ± 0.7) [21, 28]

**Fig. 6:**
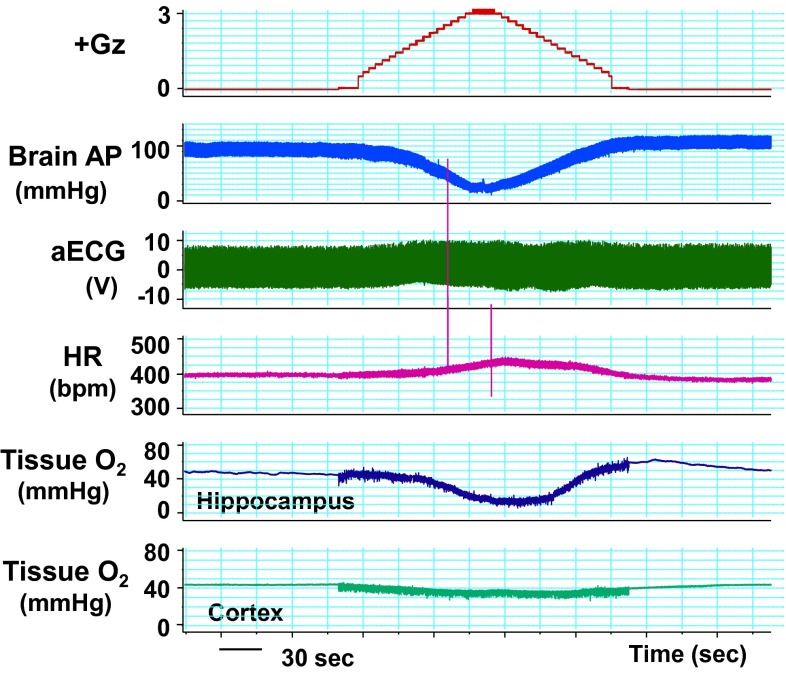
Representative original recordings of brain-tissue oxygen concentration (tissue O_2_) of the cortex and the hippocampus of a rat exposed to +3.0 Gz acceleration for 15 s without anti-G equipment. Brain-tissue oxygen concentration was measured by the oxygen monitor (multi-channel oxygen monitor, Eikou-Kagaku, Tokyo) with polarographic oxygen electrodes inserted directly to the cortex and the hippocampus. Abbreviations were same as Fig. 5. Acceleration of +3.0 Gz for 15 s without anti-G equipment decreased cortical O_2_ concentration from 61 ± 32 to 44 ± 28 mmHg, that is 71 ± 2 %, and hippocampal O_2_ concentration from 63 ± 34 to 33 ± 27 mmHg, that is 52 ± 6 %. The %-decrease in hippocampal O_2_ concentration was significantly larger than that in cortical O_2_ concentration. In addition to the decreases, the recovery time to the control level was significantly longer in the hippocampus than in the cortex. The effects of +3.0 Gz acceleration without anti-G equipment on O_2_ concentration were larger in the hippocampus than in the cortex [19, 21, 27]

**Fig. 7:**
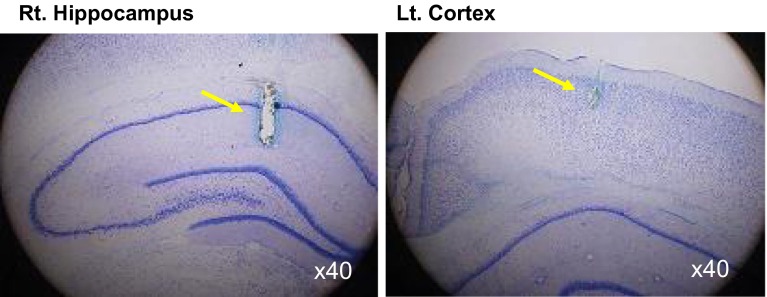
Nissl stainings of rat brain slices. *Right* the right hippocampus of a rat, *Left* the left cortex of the same rat as right. *Arrows* show the traces of the flow probe position. After the flow or tissue-O_2_ experiments, all rat brains were taken, fixed and stained to confirm the inserted position of the tissue-flow probe. The position of the tissue-O_2_ probes was also confirmed by the same way after the experiments [19–21, 27, 28]

We performed what are to our knowledge the first measurements of local brain-tissue blood flow (Fig. 5) and oxygen level (Fig. 6) of animals under +Gz load using a laser tissue-blood flowmeter (Omega Flo-C1, Omegawave, Tokyo) [21, 28] and a tissue oxygen partial pressure monitor (multichannel oxygen monitor, Eiko Kagaku, Tokyo) [19, 27], respectively. For these measurements, respectively, a glass-fiber flow probe and a polarography oxygen electrode were emplaced in accordance with a rat brain map [42] using a brain stereotaxis apparatus and attached to the cranial bone. The cerebral cortex and hippocampus emplacements were confirmed morphologically for all cases, by post-trial brain excision and slicing followed by Nissl staining (Fig. 7). Separate individuals were used for cortical and hippocampal blood-flow measurements (Fig. 5), with a single-channel instrument. Cortical and hippocampal oxygen partial pressures (Fig. 6) were measured simultaneously on the same individual, thanks to a multichannel instrument. Blood-flow and oxygen partial pressure measurements were performed on separate individuals, since the measurement point was the same for both and it was therefore not possible to insert both probes simultaneously.

**Fig. 8:**
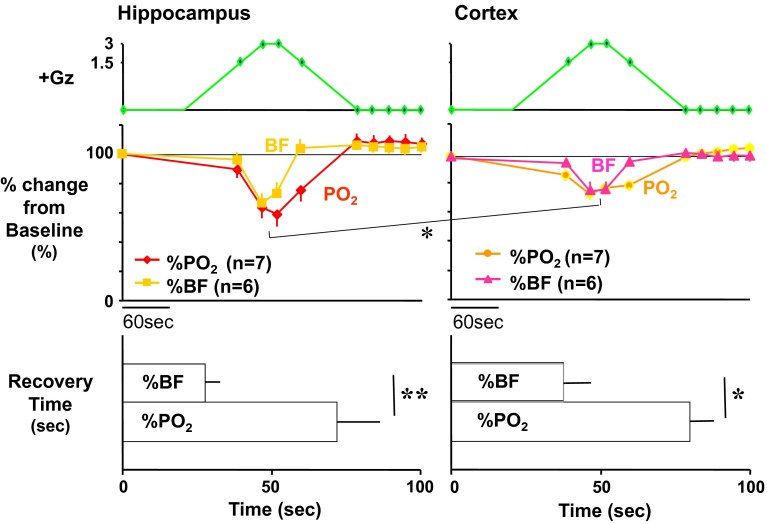
Comparisons of +3.0 Gz-induced %-changes in blood flow and O_2_ concentration between the hippocampus and the cortex. The %-changes were calculated as [measured value × 100/value in a baseline control period]. The recovery time is the duration (s) from the end of +3.0 Gz exposure to a returning point to the control level before the acceleration. Data are expressed as mean ± SE. *% BF* the %-changes in tissue-blood flow in six rats, *% PO*_*2*_ the %-changes in tissue-oxygen concentration in seven rats.*p < 0.05, and **p < 0.01 by one-factor ANOVA with the Tukey–Kramer test. Acceleration of +3.0 Gz caused significantly deeper hypoperfusion and hypoxia in the hippocampus than in the cortex, and significantly longer recovery time in *P*O_2_ than that in blood flow at both the hippocampus and the cortex [19–21, 27, 28]

Under a +3.0 Gz load, the cortical blood flow decreased from 5.7 ± 1.9 to 4.3 ± 2.3 ml/min/100 g and thus to 76 ± 9 % (*n* = 6; SE), and the cortical oxygen partial pressure decreased from 61 ± 32 to 44 ± 28 mmHg and thus to 71 ± 2 % (*n* = 7; SE). The recovery in cortical tissue oxygen level lagged behind that of blood flow by an average of 69 s. Meanwhile, the hippocampal blood flow decreased from 19.6 ± 8.1 to 13.5 ± 6.6 ml/min/100 g and thus to 69 ± 6 % (*n* = 6; SE), and the oxygen partial pressure decreased from 63 ± 34 to 33 ± 27 mmHg and thus to 52 ± 6 % (*n* = 7; SE). The recovery in hippocampal tissue oxygen level lagged behind that of blood flow by approximately 42 s. As shown in Fig. 8, the declines in both blood flow and oxygen level were significantly larger in the hippocampus than in the cortex [19, 21, 27, 28], and the times to recovery after the +3.0 Gz load were significantly longer for tissue oxygen level than for blood flow, in both the cortex and the hippocampus [20].

Under a +5.0 Gz load, though the number of cases in the experiment was small (*n* = 4), the cortical tissue blood flow and oxygen level decreased to approximately 60.0 and 26.5 %, respectively, and the hippocampal tissue blood flow and oxygen level decreased to approximately 36.6 and 23.5 %, respectively. Again, the effect on tissue blood flow was larger in the hippocampus than in the cortex with +5.0 Gz load. Comparison between the hippocampal and cortical tissue oxygen levels was difficult because both approached the lower limits.

In our research, we have not yet progressed to analysis of the relation of a decline in cerebral blood flow and oxygen level to functional impairment. We have found, however, the degree of their decline differs with the brain region (with the effect stronger in the hippocampus), and that tissue oxygen partial pressure does not recover immediately after blood flow recovery but rather generally lags behind by approximately 40 s. This lag may explain one of the causes for relative incapacitation [1] following the period of G-LOC.

We rather unexpectedly found no prior reports on animal experiments for the degree of +Gz load effects on cerebral blood flow without the use of anti-G systems. Laughlin et al. [11] found, using an anti-G system, no significant declines or changes in intra-cerebral distribution under Gz loads of +3.0 to +7.0 when using the microspheres method. We have not been able to find any prior reports on cerebral tissue oxygen level (partial pressure) distribution.

### Summary of effects without anti-G protection

The animal experiments described here showed that the physiological effects of +Gz load are far larger than generally inferred from records of human symptoms [1, 41] and other manifestations. The innate anti-G protective functions of bipedal humans presumably differ from those of quadrupeds, but the effects without any protective safety system or anti-G techniques were nonetheless surprisingly large, and we believe that true safety operations should take these effects into consideration.

## Effects of using an anti-G system

**Fig. 9:**
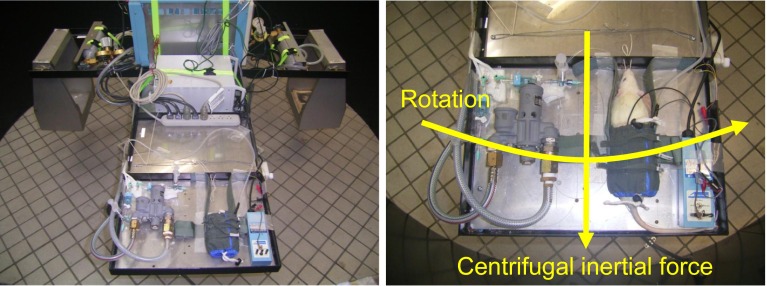
A pneumatic anti-G system developed for rats, consisted of a cylinder of compressed air, a G valve (Shimadzu Co. Ltd., Kyoto, Japan), and a rat anti-G suit (Fujikura, Parachute Co Ltd., Tokyo, Japan). The anti-G suit covered part of the rat abdomen and hind limbs, but not the tail. A G valve was placed on the table along the G axis and the level of the running weight of the G valve was positioned at a level of the rat head. The G valve runs automatically during Gz exposure and regulates the internal pressure of the anti-G suit [22]

**Fig. 10:**
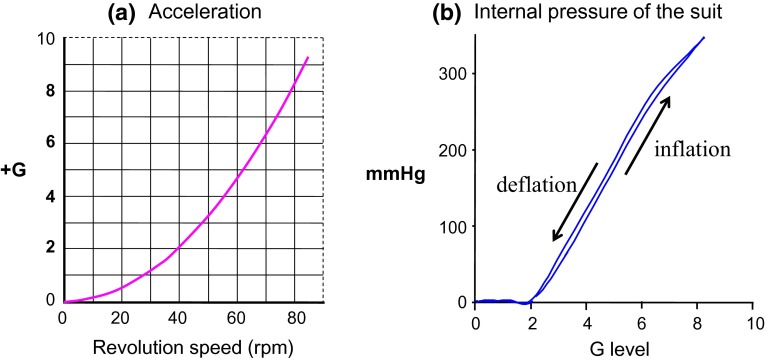
**a** A relationship between acceleration (+G) vs. revolution speed of the centrifuge (rpm). G values were determined using the length (115 cm) of the center-to-head position of the animal and revolution speed at the animal’s head. The onset or offset rate of Gz load was not linear, but rather curved exponentially with 0.03 G s^−1^ at +3 Gz, 0.04 G s^−1^ at +5 Gz and 0.05 G s^−1^ at +7 Gz [22, 31]. **b** A relationship between internal pressure of an anti-G suit (suit pressure) vs. acceleration (+G). An internal pressure of an anti-G suit was measured when the centrifuge was run up to 80 rpm. The increase of internal anti-G suit pressure started at +2.0 Gz, and was controlled depending on the Gz level: approximately 45 mmHg at +3.0 Gz, 150 mmHg at +5.0 Gz, and 255 mmHg at +7.0 Gz [22, 31]

The G-valve of the anti-G system was placed on the same flat table as the laboratory animal and secured at same level as the animal’s head with its axis lying along the Gz-axis (Fig. 9). The G-valve activated at approximately +2.0 G and automatically regulated the internal pressure in the anti-G suit in correspondence with the G load [22, 31]. The anti-G suit was designed for small animals [22, 31, 32]. It enveloped the lower body and legs, and exerted pressure circumferentially on them by increasing the pressure in a balloon installed in the suit, thus inhibiting movement of blood toward the lower body and legs. Its internal pressure was 45 mmHg at +3.0 G, 150 mmHg at +5.0 G, and 255 mmHg at +7.0 G (Fig. 10).

### Effects on cephalic arterial pressure

The effect of the anti-G system on cephalic AP was quite dramatic, and even with +5.0 Gz load it returned to approximately the value found before load application. Peterson et al. [43] and Burns [2] have reported a similar effect.

**Fig. 11:**
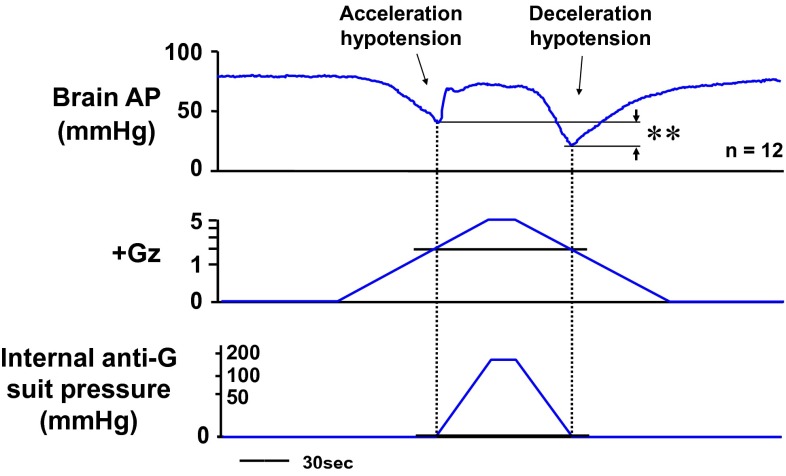
Mean responses of arterial pressure at the level of the brain (brain AP) of 12 rats with anti-G equipment exposed to +5.0 Gz acceleration for 15 s. +Gz and internal suit pressure were expressed by logarithmic scales. **p < 0.01 by one-factor ANOVA with the Tukey–Kramer test. An anti-G suit could dramatically block the +5.0-Gz-induced severe hypotension and kept the brain arterial pressure to the control level. Still, however, decreases in brain AP were observed twice; first during the increasing +Gz phase but before activation of an anti-G suit (the acceleration hypotension), and later during the decreasing +Gz phase after deactivation of an anti-G suit (the deceleration hypotension). The deceleration hypotension was significantly greater than the acceleration hypotension, although the loaded Gz and internal suit pressure during the deceleration hypotension phase were almost equal to those during the acceleration hypotension phase [17, 18, 22]. A slower onset or offset rate of acceleration in our centrifuge may permit to observe both of hypotensions, however the obtained data shows that the G-decreasing deceleration phase has more damage-full potentials built in than the G-increasing acceleration phase

In the course of our investigation, however, we were able to observe a previously unreported “double-dip” in hypotension. This observation was enabled by the slow onset and offset rates (both 0.04 G/s) used in our small-animal acceleration loading system. The first dip occurred until the G-valve was activated, and the second occurred after G-valve deactivation (Fig. 11) [17, 18, 22, 25]. We named them acceleration hypotension and deceleration hypotension, respectively [22]. Invariably, the deceleration hypotension was found to be significantly deeper than the acceleration hypotension. We were unable to find any reports on investigation or comparison of acceleration and deceleration hypotension, and more specifically on hypotension occurrence during the period of load increase from +1.0 Gz to anti-G system activation at +2.0 Gz in G-load exposure and during the period of load decrease from anti-G suit system deactivation at +2.0 Gz as the load was returned to the control value of +1.0 Gz.

To investigate this “double-dip” phenomenon, we first compared the two periods of hypotension in terms of anti-G suit internal pressure and magnitude of the Gz-load intensity. The results are shown in Fig. 11, together with AP at a level of the brain (cephalic AP). The pressure in the anti-G suit was near 0 mmHg at both time points (Fig. 10) and thus showed no difference between the two periods. The Gz load was also approximately the same at both time points, at approximately +2.2 Gz during acceleration and +2.0 Gz during deceleration (Fig. 11) [22]. The physically applied forces were thus essentially the same, but cephalic AP was significantly deeper under decreasing G load than increasing G load. We concluded that this difference in hypotension was caused by endogenous physiological responses, rather than by exogenous forces.

The difference between the acceleration and deceleration hypotension was enhanced with increasing peak Gz loads, between +3.0 and +7.0 [42], but was not affected by the duration of the peak load [22]. We therefore concluded that the severity of hypotension under decreasing +G varies in proportion with the magnitude of the G-load intensity but not its duration.

### Effects on sympathetic nerve activity

**Fig. 12:**
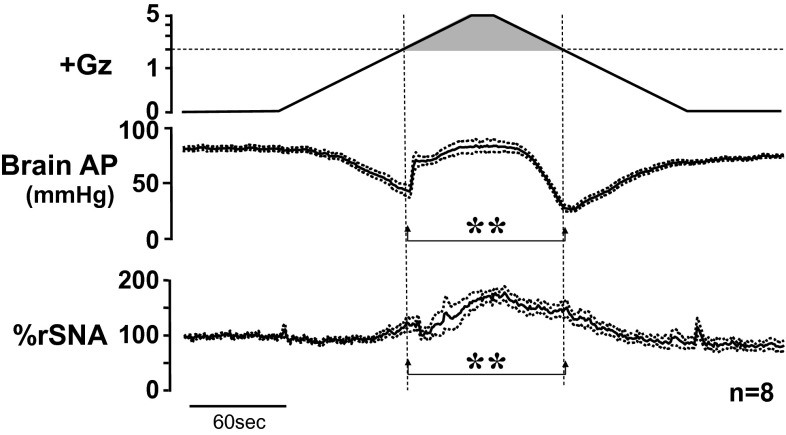
Mean responses with superimposed SE of renal sympathetic nerve activity (rSNA) with arterial pressure at the level of the brain (brain AP) of eight rats with anti-G equipment exposed to +5.0 Gz acceleration for 15 s. rSNA was expressed by % of resting activity during the control period. **p < 0.01 by one-factor ANOVA with the Tukey–Kramer test. rSNA increased at both of the acceleration and deceleration hypotensions. Increase in rSNA at the deceleration hypotension was significantly greater than that at the acceleration hypotension [17, 18, 22]. The data does not support the opinion that the deceleration hypotension may be caused by smaller responses in sympathetic activity

To investigate the causes for the difference between acceleration and deceleration hypotension, we next focused on rSNA at both times. We found that rSNA increased at both acceleration and deceleration hypotension, but the increase in rSNA was greater at deceleration hypotension than at acceleration hypotension (Fig. 12).

We have not analyzed the mechanism of the increase in nerve activity, but it may well include such factors as baroreceptor reflex, central nervous system ischemic response, and stress–defense response. Furthermore, a complex combination of overlapping responses appears to be suggested, since the peak hypotension under decreasing +G load occurs at a time when the rSNA has peaked and is beginning to decline somewhat.

Although we have not analyzed the rSNA mechanism, the occurrence of higher activity during deceleration hypertension than acceleration hypotension led us to conclude that rSNA was probably not the cause of the severe hypertension under decreasing +G load (deceleration hypotension) [17, 18, 22, 25].

We surveyed prior reports but could not find any that involved a recording of sympathetic nerve activity under Gz load, with or without an anti-G system.

### Effects on peripheral vascular resistance

**Fig. 13:**
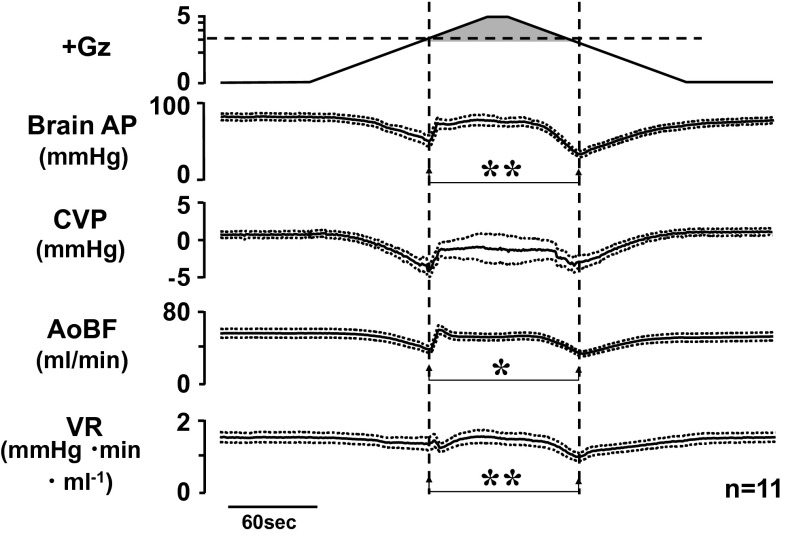
Mean responses with superimposed SE of aortic blood flow (AoBF) and total vascular resistance (VR) with arterial pressure at the level of the brain (brain AP) and central venous pressure (CVP) of 11 rats with anti-G equipment exposed to +5.0 Gz acceleration for 15 s. AoBF was measured by a transonic flow probe (2.5 mm diameter, Transonic Systems, Ithaca, NY) which was aseptically implanted on the ascending aorta 2 weeks before the experiments. VR was calculated as [(brain AP-CVP)/AoBF]. The gray area of +Gz shows the moments of anti-G suit functioning. *p < 0.05 and **p < 0.01 by one-factor ANOVA with the Tukey–Kramer test. AoBF decreased significantly greater at the deceleration hypotension than at the acceleration hypotension. VR also decreased significantly greater at the deceleration hypotension than at the acceleration hypotension [17, 18, 22]. The data indicates that one of causes for greater deceleration hypotension is due to lower peripheral resistance produced by release from squeezing pressure of an anti-G suit (based on our experimental data)

We implanted a flow probe in the ascending aorta by aseptic surgery, allowed 2 weeks or more for recovery, and then measured the ascending aortic blood flow (AoBF), which is nearly equal to cardiac output though in accurate [AoBF = cardiac output minus coronary blood flow], and compared AoBFs during acceleration and deceleration hypotension. We found that the decline in AoBF was Δ22 ± 2 ml/min during deceleration hypotension, which is significantly larger than the decline of Δ18 ± 2 ml/min during acceleration hypotension (Fig. 13) [17, 22, 25].

We also calculated the total peripheral vascular resistance (VR) by the formula [(brain-level AP minus central AP)/AoBF], and compared the values during acceleration and deceleration hypertension. The VR decline was Δ0.11 ± 0.10 mmHg/min ml at acceleration hypotension and Δ0.45 ± 0.07 at deceleration hypotension, which clearly indicates that the deceleration hypotension was caused by a decline in peripheral VR and thus by peripheral blood vessel dilation during deceleration hypotension (Fig. 13) [17, 22, 25].

A nitric oxidase synthase inhibitor, *N*-nitro-l-arginine methyl ester (L-NAME), and an indomethacin, prostaglandin synthetase inhibitor, both of which inhibit production of vasodilator factors, could not abolish the difference between acceleration and deceleration hypotension [46]. Based on the data, the dilation of the peripheral blood vessels was not caused by these local regulators, but may be related to release from the compressive effect of the anti-G suit pressure or to tissue ischemia under its compressive effect [46].

### Arterial baroreceptor reflex

**Fig. 14:**
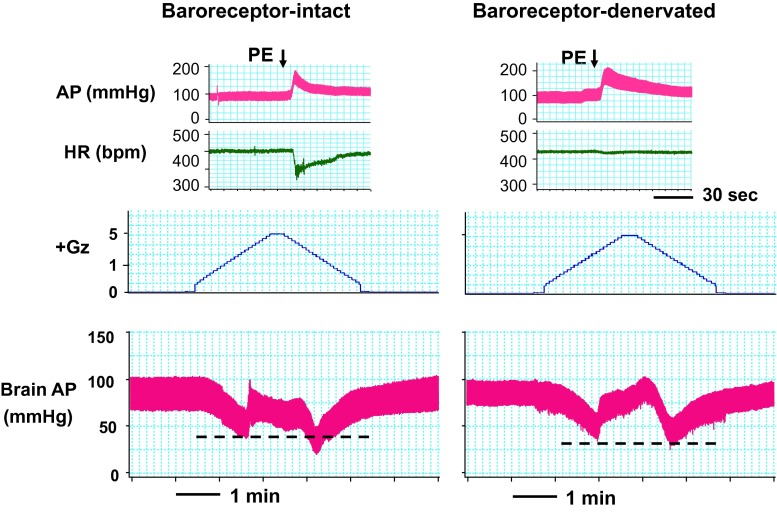
Effects of sinoaortic denervation on Gz-induced hypotensions. Typical responses in arterial pressure at the level of the brain (brain AP) of baroreceptor-intact and -denervated rats with anti-G equipment exposed to +5.0 Gz acceleration for 15 s. Sinoaortic denervation (SAD) was performed by cutting bilateral aortic nerves and carotid sinus nerves with application of 10 % phenol [6, 24]. *AP* arterial pressure at the level of the rat heart, *HR* heart rate. *Upper panels* to confirm denervation, no reflexive HR response was observed even when hypertension was induced by intravenous bolus phenylephrine (PE) in SAD rats (*Left* baroreceptor-intact, *Right* baroreceptor-denervated). *Lower panels* acceleration of +5.0 Gz induced greater deceleration hypotension than acceleration hypotension in a baro-intact rat with anti-G equipment, whereas it produced significantly deeper acceleration hypotension to the similar level of the deceleration hypotension in an SAD rat with anti-G equipment. The data from nine rats resulted in a significant difference in acceleration hypotension but not in deceleration hypotension between in intact and SAD rats, indicating that greater deceleration hypotension may be caused by failure of baroreflex function in the deceleration phase [24]

**Fig. 15:**
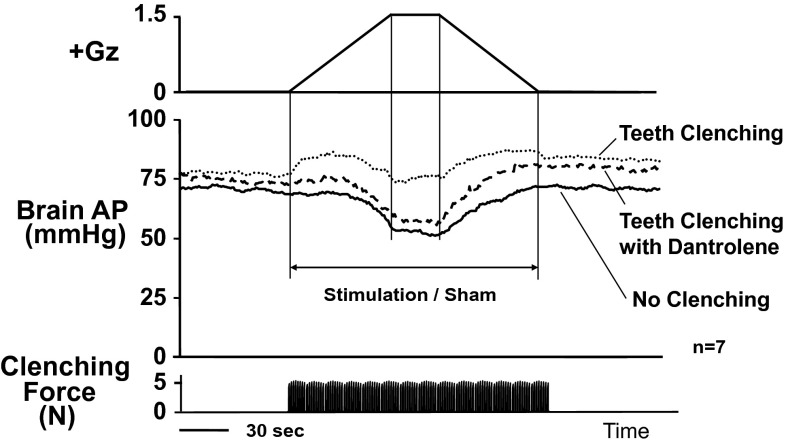
Effects of teeth clenching on Gz-induced hypotension. Mean responses of arterial pressure at the level (brain AP) of seven rats without anti-G equipment exposed to +1.5 Gz acceleration for 30 s. Teeth clenching was induced by electrical stimulation of bilateral masseter muscles of rats (20 tetanic contractions for 30 s, at a rate of 1 per s at 50 Hz for 0.4 s with pulse duration 1 ms). To measure clenching force, a 0.65-mm-thick pressure sensor (PSM-type, Kyowa Electric Instruments, Tokyo, Japan) was placed on the right lower molar teeth of rats. The electrical stimulation produced a teeth clenching force of about 5 N. Acceleration of +1.5 Gz for 30 s decreased brain AP by 18.3 ± 2.0 mmHg in seven rats without anti-G equipment when without electrical stimulation of masseter muscles (no clenching), whereas it did not significantly decrease brain AP (1.9 ± 2.0 mmHg) in the same seven rats without anti-G equipment when stimulated of masseter muscles (teeth clenching), indicating that the teeth clenching can protect the animal from the 1.5 Gz-induced hypotension. Then, dantrolene, a postsynaptic skeletal muscle relaxant, was administered to reduce masseter muscle contraction even when electrically stimulated in the same seven rats. Dantrolene markedly reduced clenching force and abolished the teeth clinching-evoked protection from 1.5-Gz-induced hypotension (decrease by 18.9 ± 2.6 mmHg for the teeth clenching with Dantrolene group) [37, 48, 49]

To investigate how and how much arterial baroreceptor reflex modulates the acceleration or deceleration hypotension, we performed arterial baroreceptor denervation (sinoaortic denervation, SAD) [6, 23, 24]. In baroreflex-intact rats, HR is lowered when AP is elevated by a pressor drug, but following SAD, HR did not decline despite AP elevation (Fig. 14, upper panels). The SAD rats were wearing anti-G suits and exposed to a +Gz load. The lower panels in Fig. 15 show a typical example of the baro-intact or baro-denervated rat, when exposed to a load of +5.0 Gz for 15 s. As shown, before SAD (*n* = 9; SD) the cephalic AP at acceleration and deceleration hypotension declined by Δ − 28.3 ± 4.0 and Δ − 46.4 ± 3.3 mmHg, respectively, and after SAD (*n* = 9; SD) the declines were Δ − 44.6 ± 3.7 and Δ − 47.9 ± 2.7 mmHg, respectively [23, 24]. With SAD, the acceleration hypotension thus deepened to approximately the same level as the deceleration hypotension. The level of deceleration hypotension, in contrast, was not affected by SAD.

Rather surprisingly, SAD was found to exacerbate acceleration hypotension but had no effect on the level of deceleration hypotension [24]. This result indicates that the arterial baroreceptor reflex function is active under increasing +G load but suppressed under decreasing +G load. Although elucidation of the mechanism must await further study, the results demonstrate that baroreceptor reflex may be suppressed once the subjects are exposed to even instantaneous +Gz load.

### Summary of effects with anti-G system

These experiments showed that even in small animals, an anti-G system can be highly effective for maintenance of cephalic AP. They also showed, however, that hypotension could occur before G-valve activation and after G-valve deactivation, and also that the hypotension after its deactivation, deceleration hypotension, is more severe. The levels of these types of hypotension are of course expected to depend on the +Gz onset and offset rates. To clarify how dependent on the onset or offset rate, further studies will be required.

With higher +Gz onset and offset rates, the hypotension would be expected to be smaller in degree, but even in this case, the possibility nevertheless remains that the effect on cephalic AP will be larger following G-valve deactivation than before its activation. At present, we have identified two causes of hypotension exacerbation under decreasing +Gz load, deceleration hypotension. One is the lowering of peripheral VR due to the release of the G-suit pressure. We did not succeed with analyses on responsible local vasodilators for peripheral vasodilatation, but we observed that hypotension similar to deceleration hypotension occurs under decreasing G-suit internal pressure when the pressure is increased and then decreased without G loading. The other is suppression of baroreceptor reflex during deceleration hypotension. In our opinion, the suppression of baroreflex may in effect be regarded as the insufficient function of a naturally endowed mechanism of defense against hypotension. In short, mechanisms are masked but exist that can exacerbate hypotension following G-valve deactivation.

We were unable to find any prior reports on animal experiments involving Gz load with an anti-G suit that included the above discussions. Several studies on other aspects have, however, been reported, particularly in regard to effects on the circulatory system. Laughlin et al. [13] showed that the left atrial pressure increases and that the renal blood flow and particularly the renomedullary blood flow decrease dramatically with +3.0, +5.0, and +7.0 Gz load in minipigs wearing anti-G suits. Whinnery et al. [53] reported that in minipigs under similar conditions, the right ventricular pressure increases. Laughlin et al. [12] reported that the left ventricular pressure increases in their description of the use of the microspheres method in baboons to investigate blood flow to various organs under a load of +5.0 Gz. They found that when the baboons wore an anti-G suit, the blood flow to the brain and the heart remained more or less unchanged but the blood flow to the kidney, spleen, and pancreas decreased dramatically. In a separate report [14], Laughlin et al. describe their investigation of the small effect on coronary blood flow, in which they closely examined that flow and verified that it increases somewhat at +5.0 Gz. These reports indicate that use of an anti-G system maintains cephalic AP and cerebral blood flow, that coronary circulation corresponds well despite increases in intracardiac pressure and cardiac work, and further that abdominal organs are subjected to ischemia.

Taken together, our experiments showed that there is a danger of cerebral hypoperfusion just before and just after the anti-G system is active, with the danger being strongest after its deactivation, when the hypoperfusion tends to be deepened by dilation of the peripheral blood vessels, suppression of baroreceptor reflex, and other factors.

## Trials to protect the brain autonomically

Our experiments showed that the effects of +Gz load on the brain without use of an anti-G system are greater than previously recognized, and that even the use of anti-G equipment involves potential of a particular danger of deepened hypotension during relaxation of the anti-G suit compression. These facts led us to the question of whether there exists some means of protecting the brain from these effects, whether with or without an anti-G system. In the effort of finding and developing methods of preserving safety under +Gz load, we found two means possibly to protect the brain from hypoperfusion.

### Teeth-clenching pressor response

We investigated the effects of induced teeth clenching on AP in rats by electrically stimulating their masseters. We placed small electrodes on the surface of the left and the right masseter muscles and applied a square-wave 7–8 V electric potential of 0.4-s duration once a second for 30 s. We measured the teeth-clenching force throughout that period with small pressure sensors inserted between the upper and lower molars, which showed a constant force of approximately 5 N. During the same period, AP increased by Δ12 ± 2 mmHg and HR concurrently increased by Δ13 ± 4 bpm [14]. Similar electrical stimulation of the femoral muscles induced no change in AP or HR. Following systemic administration of a postsynaptic skeletal muscle relaxant, dantrolene, the masseter electrical stimulation induced no appreciable increase in AP or HR [48]. These findings indicated that the pressor response under masseter contraction was specifically induced by the teeth-clenching action, and we accordingly named it the “teeth-clenching pressor response” [48]. The receptors and reflex mechanisms for the pressor response are now investigating and be on the way [49].

When we applied +1.5 Gz load to the rats together while inducing the teeth-clenching action by the electrical stimulation of their masseters and thus inducing the teeth-clenching action, we found that Gz-induced cephalic hypotension was negated in proportion to the AP increase induced by the teeth clenching (Fig. 15). The results showed that the teeth-clenching action led to an anti-G effect equivalent to a reduction in Gz load of approximately 1.5 Gz [48].

We are currently pursuing investigations into the optimum frequency of teeth clenching for the most effective pressor response, and the physiological mechanism (e.g., the stimulus signals, afferent nerves, and efferent output) [44]. Studies are also desirable in relation to a mouthpiece and other related developments for practical utilization of the teeth-clenching pressor response.

### Preconditioning effects

We tried to investigate the possibility of obtaining a heightened endogenous autonomic G-tolerance, prior to regular normal acceleration norms and training, through multiple repeated exposures to Gz load that has minimal or weak effect on the brain but may induce some physiological adaptable responses [29, 45].

Exposure to +1.5 Gz load resulted in a decline of 0.2 ± 0.2 mmHg in CVP and 18 ± 2 mmHg in cephalic AP of anesthetized rats (*n* = 15; SD). These small declines due to this comparatively light Gz load induce virtually no degradation in cerebral function. We performed the +1.5 Gz load every second day for 15 min three times per day on 5 days, and thus a total of 15 light +Gz loads. The rats in the group that had undergone this conditioning were subsequently exposed to a load of +3.0 Gz for 30 s, and we measured the resulting changes in brain AP, cerebral cortex tissue oxygen level (cortical *P*O_2_), and hippocampus tissue oxygen level (hippocampal *P*O_2_) and compared the changes in these three parameters with those of the rats in the non-conditioned group.

**Fig. 16:**
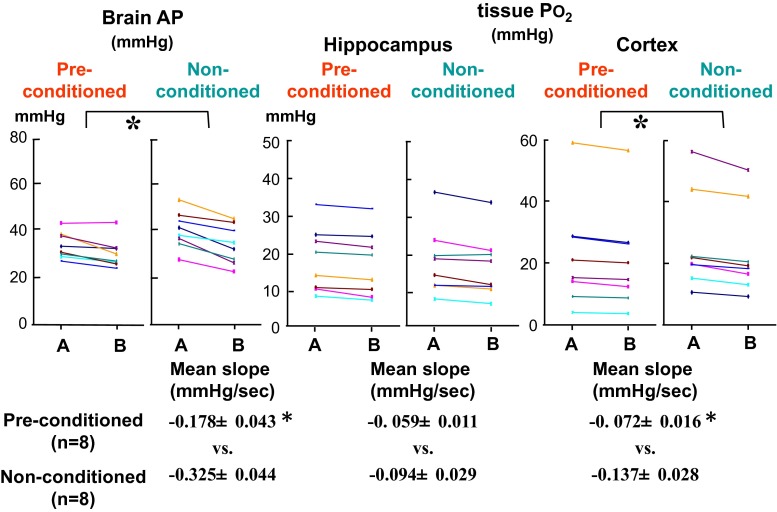
Effects of preconditioning on Gz-induced cerebral hypoperfusion. Two weeks before the acceleration stress of +3.0 Gz for 30 s, rats (*n* = 8) were exposed to repeated lighter Gz acceleration stress (+1.5 Gz for 15 min, three times per day, one of 2 days, five cycles). Three days later after the conditioning, the effects of +3.0 Gz acceleration for 30 s on arterial pressure at the level of the brain (brain AP) and brain-tissue oxygen concentration (tissue *P*O_2_) were measured in the conditioned rats (Pre-conditioned, *n* = 8), and compared to those in non-conditioned control rats (non-conditioned, *n* = 8). Each of 8-rat data were presented. *p < 0.05 by one-factor ANOVA with the Tukey–Kramer test. The decreasing rate (*Slope* decrease/s) in PO_2_ of the hippocampus during 30 s of +3.0 Gz did not show any significant difference, whereas the slope in APLB and *P*O_2_ of the cortex during the 30 s showed significant differences between conditioned and control rats [26, 29, 37]

No significant difference was found between the conditioned (*n* = 8) and non-conditioned (*n* = 8) groups in any of the three parameters just after attainment of the +3.0 Gz load, but two of the three were found to have declined significantly further in the non-conditioned group than in the conditioned group 30 s after the attainment of +3.0 Gz, and thus during the 30 s at +3.0 Gz (Fig. 16). Significant differences between the two groups during the +Gz load were found in both cortical and hippocampal *P*O_2_ but not in cortical *P*O_2_. Preconditioning (repeated lighter G stress) before the heavy G stress was thus found to eliminate the responses of cerebral AP and cortical oxygen decline under 15 s at a load of +3.0 Gz that were observed in the non-conditioned group [26, 29, 34, 37, 45].

In cerebrovascular endothelial cells, NO acts as a local circulatory regulator [13] to increase the tissue-cGMP concentration. We compared the cerebral-tissue cGMP concentrations in the conditioned and non-conditioned groups, as a possible indicator of intracerebral molecular transformation induced by the conditioning, but unfortunately found no significant difference between them [26, 34, 37].

In our research, although we were thus unable to show that the effects of conditioning reflect intracerebral molecular changes, we were able to show its functional effects, in the form of prevention of cephalic AP and hippocampal oxygen level decline during 30 s under a +3.0 Gz load. In this way, we showed that repeated exposure to light +Gz load that does not result in degraded brain function can induce a tolerance for +3.0 Gz load. Of course, the effects were slight, but the results indicate that it may be possible to obtain greater effects through refinement of the preconditioning method.

## Conclusions

The results of these animal experiments on +Gz load suggest the possibility that the effects of +Gz load on the brain and the heart may be far larger than previously inferred from investigations with human subjects. Naturally, inter-species differences are prevalent throughout nature and no doubt exist between bipeds and quadrupeds, but it is nevertheless important to note that the decrease in cerebral blood perfusion pressure to near-zero and subtle myocardial damage at +5.0 Gz discovered in these animal experiments were unforeseen.

Anti-G systems provide strong protection of the brain from cephalic hypotension and protection of the heart from myocardial damage. As shown by these experiments, however, their use also may involve a relatively strong effect on cephalic arterial perfusion pressure before G-valve activation and after its deactivation. The experimental results indicate in particular that the relaxation of compression by the G-suit following its deactivation may possibly pose the larger danger.

The experimental results also revealed the possibility of minimizing damage by +Gz load through increasing the G-load tolerance of the physiological autonomic or involuntary functions. The known methods of increasing G-load tolerance have generally involved utilization of physiologically voluntary mechanisms (e.g., skeletal muscle contraction and the voluntary Valsalva maneuver). In our research, in contrast, we have focused on the possibility of methods that can increase G-load tolerance through involuntary, autonomic physiological functions. One method that we investigated is the teeth-clenching pressor response, and another is a method of preconditioning. Practical application of the teeth-clenching method would be relatively simple. The preconditioning method, on the other hand, may provide a starting point for development of a scientifically based regimen of acceleration training.

Cumulative research over an extensive time span will be necessary to elucidate the changes induced in long-term mechanisms of blood-pressure regulation [38–40] and short-term circulatory functions by these effects of +Gz load and the trained G-load tolerance. Understanding endogenous physiological responses will in turn provide new information and materials leading to further investigations. Animal experiments within the scope provided by the ethical code enable research that simply cannot be conducted on human subjects, and may be assumed as vital for the attainment of true knowledge and understanding rather than inference and supposition.

In the present short-review, we provide the data for biological limitations/causinos, and possible measures to heighten an endogenous autonomic G-tolerance. The former is that when without any protection against Gz stress, (1) +5.0 Gz stress causes zero-pressure in cephalic artery and minus 10 mmHg in CVP, (2) +3.0 Gz stress causes sympathetic withdrawal response, baroreflex dysfunction, arrhythmia, myocardial cell injuries, heterogeneous brain perfusion, and delayed recovery from brain hypoxia. Even with anti-G systems, hypotension occurs twice before and after the start of G-valve activation, which are acceleration hypotension and deceleration hypotension. Deceleration hypotension is more dangerous than acceleration hypotension due to functional abrasion of baroreflex and vascular incontractility. As possible measures to heighten endogenous autonomic G-tolerance, teeth clenching with mouse pieces and preconditioning training are proposed, which means that the organisms can adapt to the G-stress somewhat.
